# Policy 2.0 Platform for Mobile Sensing and Incentivized Targeted Shifts in Mobility Behavior

**DOI:** 10.3390/s16071035

**Published:** 2016-07-05

**Authors:** Ivana Semanjski, Angel Javier Lopez Aguirre, Johan De Mol, Sidharta Gautama

**Affiliations:** 1 Department of Telecommunications and Information Processing, Ghent University, St-Pietersnieuwstraat 41, Ghent B-9000, Belgium; angel.lopez@ugent.be (A.J.L.A.); johan.demol@ugent.be (J.D.M.); sidharta.gautama@ugent.be (S.G.); 2 Facultad de Ingeniería Mecánica y Ciencias de la Producción, Escuela Superior Politécnica del Litoral, ESPOL, Campus Gustavo Galindo Km 30.5 Vía Perimetral, Guayaquil 09-01-5863, Ecuador

**Keywords:** Policy 2.0 platform, smart cities, sustainable mobility, mode shift incentive, travel behavior, smartphone mobility applications

## Abstract

Sustainable mobility and smart mobility management play important roles in achieving smart cities’ goals. In this context we investigate the role of smartphones as mobility behavior sensors and evaluate the responsivity of different attitudinal profiles towards personalized route suggestion incentives delivered via mobile phones. The empirical results are based on mobile sensed data collected from more than 3400 people’s real life over a period of six months. The findings show which user profiles are most likely to accept such incentives and how likely they are to result in more sustainable mode choices. In addition we provide insights into tendencies towards accepting more sustainable route options for different trip purposes and illustrate smart city platform potential (for collection of mobility behavior data and delivery of incentives) as a tool for development of personalized mobility management campaigns and policies.

## 1. Introduction

Recently smart cities have become one of most investigated research topics across different fields. Their main component is the use of advanced sensing techniques and the integrated Internet of Things (IoT) concept to facilitate smarter and more sustainable living in city areas. In this context, transportation has a lot to contribute as it generates about 25% of global CO_2_ emissions [1]. In addition, just urban mobility accounts for 40% of all CO_2_ emissions of road transport and up to 70% of other pollutants from transport [2]. Therefore the achievement of smart cities’ goals is inevitably related to advances within the scope of sustainable mobility.

In this sense, different policy options have been designed with the aim to promote more sustainable mobility behavior [3]. Nevertheless studies show that these policies have had limited effect as people merely reacted to given constraints while their intrinsic motivation failed to be triggered [3,4,5]. Psychological studies took more detailed look [6,7] and have suggested that the reason for this can be found in fact that policy options target whole populations in a uniform way. Thus, without deeper understanding of individuals’ attitudes, values or motivation, they are failing to trigger the desired behavioral changes. In line with this indication, Schade and Schlag [8] showed that social norms, personal outcome expectations and perceived effectiveness are positively related with acceptability of urban transport pricing strategies and that they account for nearly 40% of the criterion variance and thus can explain acceptability of such measures much better than socio-economic variables. Another study [9] showed that knowledge about personal motives is essential for further growth of car-sharing usage. Beirao and Cabral [10] and Shiftan et al. [11] examined the role of travelers’ attitudes and its relation towards the choice of transport, while Nkurunziza et al. [12] and Li et al. [13] showed that personal motivation and attitudes are strongly associated with bicycle commuting. Therefore, in order to deliver more effective sustainable mobility related policy measures, transfer of market segmentation techniques in the field of mobility has been explored [6,14] Market segmentation techniques aim to reduce the complexity and heterogeneity of whole populations by dividing them into relevant subgroups for which then targeted and personalized mobility management campaigns and policies can be developed. Furthermore, an attitude-based segmentation approach seems particularly applicable in this context as the resulting subgroups exhibit higher homogeneity in terms of attitudes (individual’s feeling of favorableness or unfavorableness) towards different options. This approach showed great potential and a number of studies [15,16] have applied attitude-based segmentation with the aim to focus their efforts in promoting more sustainable mobility behavior for one of the targeted subgroups. Some practical examples of this include attempts to impact mobility behavior for new residents in Sofia, Utrecht, Munich and Almada [17] or for school and university students in Hounslow, Sofia, Utrecht, Gdynia, Munich and Almada [18,19]. Nevertheless, to best of our knowledge, so far there are no empirical studies that evaluate the effect of attitude-based segmentation techniques in regard to more sustainable mobility behavior at a holistic level.

The research mentioned above included dedicated workshops, competitions between classes, cycling practices and handouts in order to communicate targeted messages while the segmentation itself was based on data collected by paper surveys. Nevertheless, today ubiquitous smartphones have become an attractive option for large-scale sensing of human behavior [20,21,22] for smart city oriented applications. Foremski et al. [23] showed that smartphones can be used for crowd sensing with a decrease in battery lifetime by approximately 20%, which they found to be acceptable by users. Furthermore, smartphones are used as precise indoor positioning sensors in order to improve intelligent parking service [24] or as activity recognition sensors [25,26]. Wan et al. [27] proposed a use of mobile crowd sensing technology to support creation of dynamic route choices for drivers wishing to avoid congestion and Xia et al. [28] explored the use of smartphones, as sensors, for detection of transportation modes from users’ movement data. In addition, smartphones can also be used as a communication channel to deliver incentives in order to trigger change in one’s mobility behavior [29,30]. So far the use of smartphones to deliver personalized messages has been explored in the field of mobile marketing [31]. Chen and Hsieh [32] tried to identify important attributes in designing a mobile advertising message for products (goods and services) and explore potential applications of these attributes. Tang et al. [33] explored the use of location information and location- related context data in predicting customers’ preferences and Watson et al. [34] examined consumers’ point of view on mobile marketing. However, all of these studies are related to advertising of products and are based on observed behavior, location-related, data without: (i) taking a deeper look into ones’ attitudes or motives and (ii) focusing on the commercial aspect of the delivered messages. Compared to these, our study examines the potential of smartphones as communication channels to deliver personalized incentives, which are primarily related to ones’ attitudes, values and motivation, in order, not to sell products, but to trigger more sustainable mobility behavior. This way, the final outcome of the delivered incentive is a desired behavioral change from which only users and their communities will derive a benefit (not commercial third parties) which is a major difference in regard to existing mobile marketing related research. When considering the use of smartphones in combination with delivery of personalized incentives for mobility relevant purposes, related studies are mainly focused on use of gamification as an appealing element that keeps user loyal to the application and thus ensures a communication channel between the incentive provider and the user itself. In this context they rely on player types as a segmentation approach where the population is divided based on their responsivity to different game mechanics. An in-depth analysis into relation of player types in regard to ones’ attitudes towards sustainable mobility options and motivation to change mobility behavior itself is absent in these studies and still remains to be explored [35].

Extending the current research on smart city mobility applications, and in the meantime addressing the above mentioned limitations, the aim of this study is to explore how innovative sensing theologies can be used in incentivizing targeted shifts in mobility behavior and to investigate how mobility system users are most likely to adopt more sustainable mobility behavior, based on the incentives provided via smartphone devices, in order to evaluate the applicability of the theory of market segmentation in urban social marketing. Furthermore, our study, to the best of our knowledge, provides first empirical results on use of smartphones to deliver personalized mobility route suggestions and evaluate the impact of this incentive on targeted shifts in mobility behavior**.** The fundamental research contributions of this work can be situated in the following areas: (i) we explore the use of smartphones as sensing devices for a smart city oriented platform; (ii) we extend the current level of knowledge on transportation system users’ attitudinal profiles by providing detailed mobile sensed description of their observed mobility behavior; (iii) we, to the best of our knowledge, provide first quantified insights into responsivity of different attitudinal profiles to incentives delivered through mobile phones; (iv) we report the first empirical results on more sustainable mode choice tendencies for different attitudinal profiles based on the use of smartphones for sensing human behavior; (v) we illustrate the smart city platform potential (for mobility behavior data collection and incentives delivery) as a tool for development of personalized mobility management campaigns and policies.

The rest of this paper is organized as follows: Section 2 defines the conceptual framework of our study. Adopted attitudinal segmentation approach, collection of mobility behavior data and delivery of incentives to users are described in Section 3. In-depth analysis on observed behavioral change is given in Section 4. Section 5 provides discussion and identifies future research needs on this topic. Finally, Section 6 gives a summary of major conclusions.

## 2. Conceptual Framework

The present research uses the theory of planned behavior [36] as a framework for attitude-based segmentation in investigating how mobility system users can be incentivized to adopt a more sustainable mobility behavior via smartphone devices. The theory of planned behavior proposes that behavior is proximally caused by intention to perform that behavior, and person’s perceived behavioral control. The perceived behavioral control is the extent to which a person believes that the behavior in question is under his or her control and it influences behavior both in direct and indirect way (through intention). In turn, intention is influenced by three conceptually independent determinants. The first one is the person’s attitude (A) toward the behavior. The attitude refers to the sum total of a person’s beliefs (b) about the outcomes of the behavior under consideration, weighted by the subjective evaluation (e) or importance one attaches to those outcomes (favorable or unfavorable evaluation of the behavior in question). A person’s attitude (A) is seen as directly proportional to this summative belief index. The second predictor is a social factor termed subjective norm (SN). The subjective norm refers to the perceived social pressure to perform or not to perform the behavior in question. Therefore SN is seen as directly proportional to the sum of normative beliefs (nb) (likelihood that important referent individuals or groups approve or disapprove of performing a given behavior) and person’s motivation to comply (c) with the referent in question. The third one is the degree of perceived behavioral control (PBC). This factor refers to the perceived ease or difficulty of performing the behavior in question (p) and it is assumed to reflect past experience as well as anticipated impediments and obstacles (cb). Following the provided descriptions, the theory of planned behavior describes the behavioral intention (BI) as:

(1) $$BI=\;w_{1\;}\overset{n}{\underset{i}{\sum }}b_{i}e_{i}+\;w_{2}\overset{n}{\underset{i}{\sum }}nb_{i}c_{i}+w_{3}\overset{n}{\underset{i}{\sum }}p_{i}cb_{i}$$

where $w_{i}$ are empirically derived weights. As a general rule, the more favorable the attitude and subjective norm with respect to a behavior, and the greater the perceived behavioral control, the stronger should be an individual’s intention to perform the behavior under consideration. Therefore, the central premise of the theory of planned behavior can be summarized as that the sequence leading from beliefs to behavior is a rational process in which individuals systematically examine, process and utilize the information available to them to arrive at a behavioral decision. For this reason the theory of planned behavior is considered to belong to the rational choice models group.

In our study, we proved users with timely information about alternative routing and transportation mode options, that is in line with their attitudes, subjective norms and perceived behavioral control. This way they can incorporate this information in their behavioral decision- making process. Further, we evaluated if provided incentive (information) actually impacted their behavioral decision-making process or they maintained their habitual behavior despite the available information. In this sense, we evaluate how the theory of planned behavior is applicable in urban social marketing regarding activities that are considered to be highly habitual [37,38] and how this method can effectively be used, in synergy with innovative sensing technologies, by policy makers to encourage more sustainable mobility behavior at the individual and community level.

## 3. Method and Data

The data collection process was a part of sustainable mobility campaign conducted in the area of city of Leuven, Flanders and lasted from January 2015 to June 2015. The campaign aimed, among other objectives, to develop a smart city mobility platform that could be used as a tool for policy makers to collect data on mobility behavior and develop targeted and more efficient mobility options. In this context, we investigated the potential of smartphones, as ubiquitous sensing devices, for collection of mobility data and delivery of personalized information in order to motivate users to explore alternatives and take more informed and more sustainable mode or route choice decisions. To achieve this we applied attitudinal segmentation approach developed under the European Segment project [39]. The Segment project explored the use of consumer market segmentation techniques, particularly the theory of planned behavior, in persuading people to change their travel behavior and adopt more energy-efficient forms of transport. The main aim of the project was to develop a transferable market segmentation model and methodology that would be replicable and transferable to all European Union member states. During the project’s lifetime (from April 2010 to April 2013) the developed approach has been tested and successfully implemented in six partner cities for at least one of the targeted user groups. As measured by monitoring surveys against collected baseline data in each city, the results have led to changes in public attitudes (perception, satisfaction, familiarity, favorability, aspirations, values) and behavior towards sustainable transport modes. Therefore, the aim of the adopted segmentation approach, in our study, was to group mobility system users in one of eight profiles (predefined by the Segment project) based on their attitudes towards sustainable mobility options, climate change and health in order to deliver targeted mobility information.

### 3.1. Attitudinal Segmentation

To participate in the research one was required to register via a dedicated web portal [40] and download a freely available smartphone application for the Android platform developed at Ghent University [41]. During registration process participants were able, on a voluntary basis, to fill out a ‘Golden questions’ survey [42]. The ‘Golden questions’ are the smallest set of survey questions (produced based on more than 10,000 questions from more than 100 surveys used during Segment project) that discriminate the most among different segments of transportation system users based on their attitudes towards sustainability [39]. They have been developed for practical reasons, in order to avoid the use of a full set of questions, and can reproduce market segments previously created from longer lists of questions with high accuracy [42]. After filling in the ‘Golden questions’ survey, based on answers provided, users were assigned one of eight predefined attitudinal profiles. These attitudinal profiles include [43]:

- Active Aspirers—They have a high moral obligation to the environment and believe that by making responsible mobility decisions they can make a difference. They feel guilty when using their car for short journeys and are highly motivated to use active transport modes. They predominantly cycle as they find it to be quick and to provide freedom and fitness. They are not likely to use public transport, but they do like to walk and would even like to walk more for fitness.
- Car-free Choosers—They consider that cars lead to unhealthy lifestyles and do not like to drive. They prefer cycling as they feel a high moral obligation to the environment. Alternatively, they will choose public transport, which they do not consider to be stressful nor problematic, and walking. They are more likely to be women.
- Car Contemplators—They do not use car but intend to as they see it as status symbol. They have high proportion of students and the highest proportion of non-driving license owners. They prefer public transportation over cycling, but do see a lot of problems with the use of public transportation and find both public transport and cycling to be stressful. They believe walking is healthy and have a neutral or moderate attitude towards the environment.
- Devoted Drivers—They use car frequently and have no intention of reducing car use. They do not see themselves as the kind of person that would use public transportation (find it stressful), bike or walking (consider it to be too slow). They have a very low moral obligation to the environment and are not motivated by fitness.
- Image Improvers—They enjoy driving and see it as a way of expressing themselves. They do not intend to reduce car use but are open to cycling and maybe walking. They think cycling can be a form of self-expression and are motivated by fitness to use it. They see walking also as a way to stay fit, but find it to be very slow. Use of public transportation is the least favorable option for them. They have neutral or moderate environmental attitudes.
- Malcontent Motorists—They do not like to drive and find it stressful. They have moderately strong intention to reduce car use but are not motivated to increase the use of public transport, although they prefer it more than cycling. They walk, but do not see any advantage to walking, except for fitness. They have a small level of environmental consciousness.
- Practical Travelers—They are confident that they are using a balanced amount of each transportation mode. Therefore they do not intend to reduce car use, although they think it reduces the quality of life, as they believe they are using it only when necessary. They prefer cycling over the use of public transportation as they consider it to be quicker and not stressful. They walk when it seems more practical than cycling and see local pollution and congestion as issues but are not motivated by climate change. They are highly educated and above-average part-time working.
- Public Transport Dependents—They do not like driving but think people should be allowed to use cars as they please. They would like to see less congestion and consider investing in more road infrastructure to be appropriate solution. They walk and would like to walk more for fitness. They also use public transport, although they think that it is not the quickest method but they prefer it more than cycling. They see no benefits to cycling and find it to be stressful. They are not motivated by the environment, are least likely to start driving and include the highest number of retired people.

**Figure 1:**
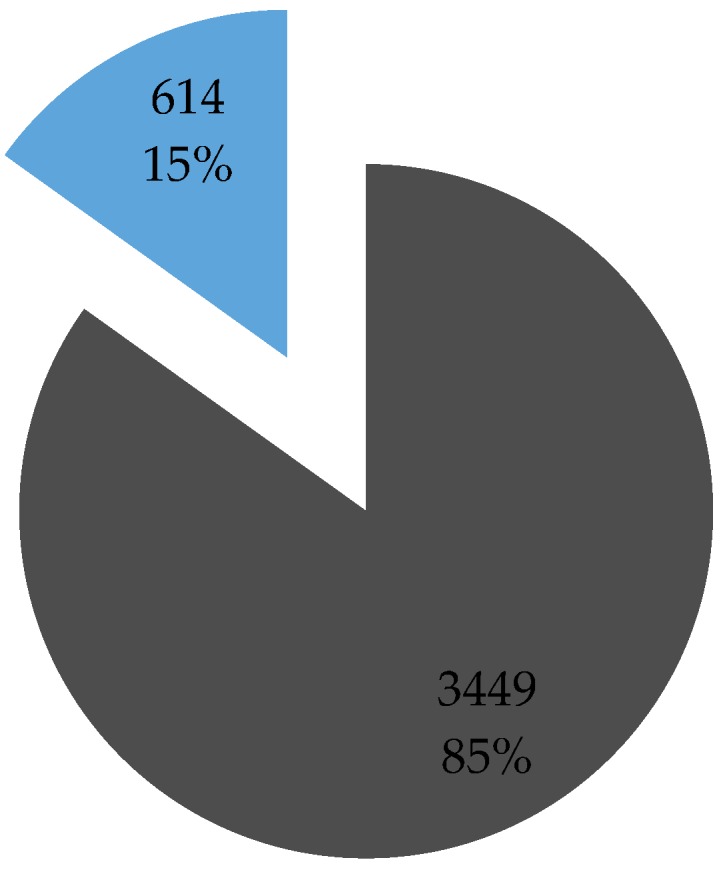
Survey response rate.

**Figure 2:**
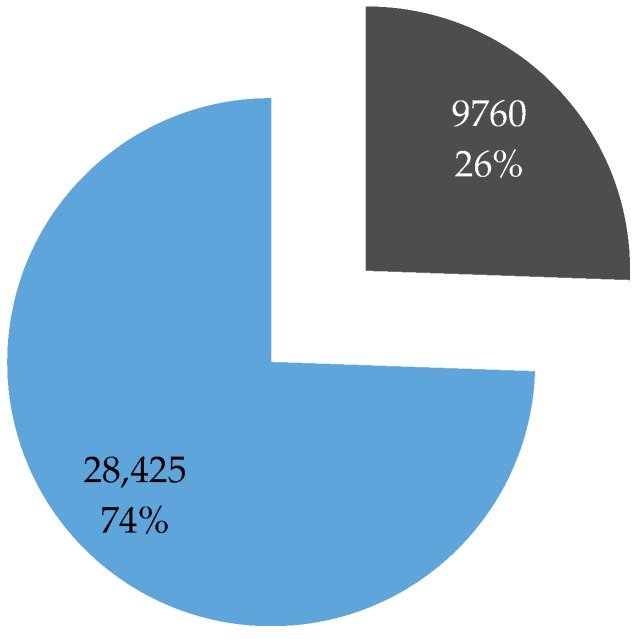
Trips—participation level.

The response rate to the ‘Golden questions’ survey was around 15% (Figure 1). This percentage is achieved by comparing the overall number of users who downloaded the app/registered for at least one trip and those who completed the ‘Golden question’ survey. Literature reports [44] that response rates to on-line surveys are lower than those to conventional paper surveys and for the general public they range between 1% and 20% [45]. The users who filled in the ‘Golden questions’ survey were also those who participate most actively in mobility behavior data collection providing almost three quarters of all collected data on undertaken trips (Figure 2).

**Figure 3:**
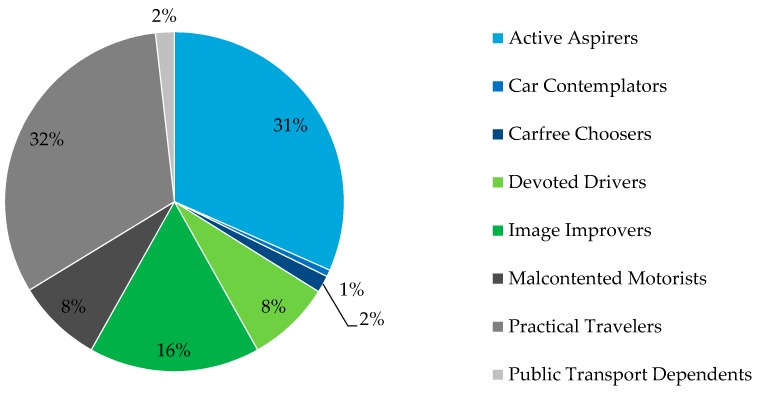
Users per attitudinal profile.

Based on the ‘Golden questions’ survey results, the most represented attitudinal profiles in our sample were Practical Travelers and Active Aspirers and the least Car Contemplators (Table 1) (Figure 3).

**Figure 4:**
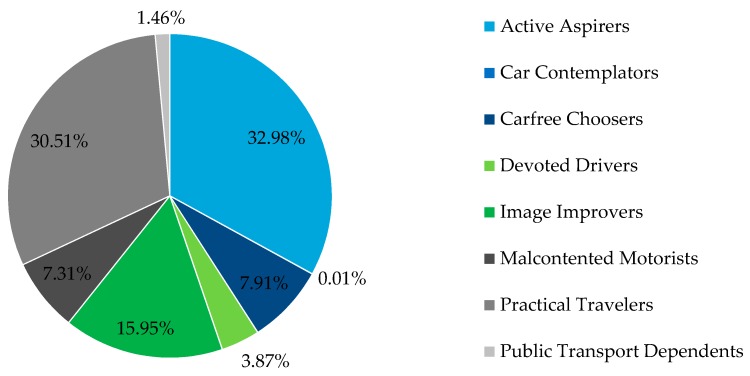
Trips per attitudinal profile.

During the data collection period Carfree Choosers collected the most trips per user (in average, 225 trips) and Car Contemplators and Devoted Drivers the least. All other attitudinal segments had similar averages, between 38 and 48 trips per user, resulting in quite proportional ratio of participants per attitudinal segment and collected trips per attitudinal segment (Figure 4).

### 3.2. Mobility Behaviour Data Collection

**Figure 5:**
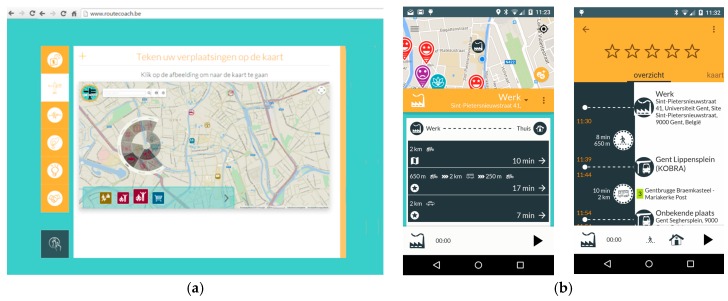
Web portal and mobile application; (**a**) Web portal; (**b**) Mobile application.

As mentioned in the Section 3.1, we used a web portal [40] and smartphone application [41] to collect mobility behavior data. Through the web portal (Figure 5) users were able to enter his/her usual points of interest (e.g., home and work location), usual routes (e.g., commuting by bike), trip purposes (e.g., shopping, recreation activities etc.), monitor personal or overall statistics and perform quality control on collected data. The mobile app was used as a routing hub that based on the user- generated requests collected routing information between defined origin and destination points. The routing information was collected from multiple sources on different modes (e.g., train route information from the national rail company, public transportation routes from public transportation company, etc.) providing users with possible routes by single transportation mode (car, bike, foot, train or public transport) as well as any feasible combination of multimodal routes (Figure 5). The origin and destination points could be indicated by a user through the map (or address) entry or simply by recalling the usual points of interest that were previously annotated through the web portal. Order of the route suggestions (the response to the routing request) was heuristically personalized based on the preferences for attitudinal profile to which the user belonged (as defined in Section 3.1). An additional element impacting the routing results order was a considered sustainability of the mode choice option (e.g., active transportation modes were considered as more sustainable option than car). For example, for the route request generated by the user matched to the Carfree Chooser profile, and for in-city trips, the first suggestion would include a bike route (the most preferred mode choice by Carfree Choosers and the sustainable one), followed by the second best bike route (the first route suggestion involving active transportation mode would be additionally highlighted by providing an additional, second best, option), shared bike route, walking route, any combination of these and public transportation routes. For routing requests over a longer distance, the first suggestion would include bike route (if shorter than 45 min—an empirically-based limit for experienced bikers [46,47]), second best bike route, shared bike route (same duration limit), public transport route (if feasible), train route (if feasible) or any multimodal combination of previously mentioned transportation modes. In a case there were no feasible sustainable routes between the origin and destination the user would be provided with information on a bike (or walking) distance route to a car sharing point and shared car route. Finally, the car route option would be suggested. Public transportation and train routes were limited to at most two transfers within the city and three transfers for long distance route requests [46]. Overall, first five feasible route suggestions would be displayed to the user who would then mark the one he would use. Same level of personalization was provided for other attitudinal segments with a fixed rule that car route could not appear as the first suggestion. This would mean that even car-oriented profiles would always receive at least one other mode choice suggestion before a car route (e.g., Image Improvers would receive a bike route suggestion first).

**Figure 6:**
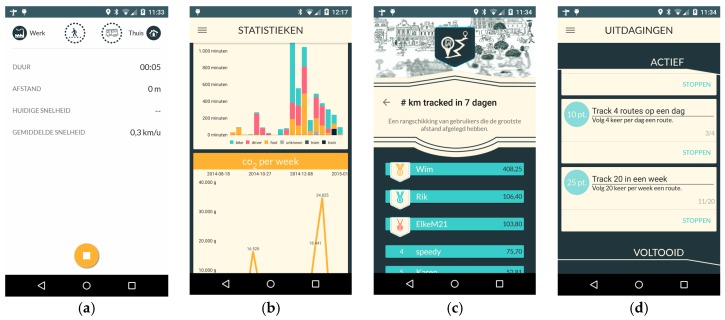
Self-monitoring and social activities; (**a**) Undertaken route information; (**b**) Personal statistics; (**c**) Leader boards; (**d**) Challenges.

The smartphone application also provided users with self-monitoring possibilities (e.g., a user could see his/her modal split, level of physical activity monitored based on the burned calories and CO_2_ and particulate matter (PM) emissions) and social boards (e.g., leader boards for the most biked kilometers or a sustainability challenge board where friends could challenge each other to, for example, walk more kilometers during a week) (Figure 6).

**Figure 7:**
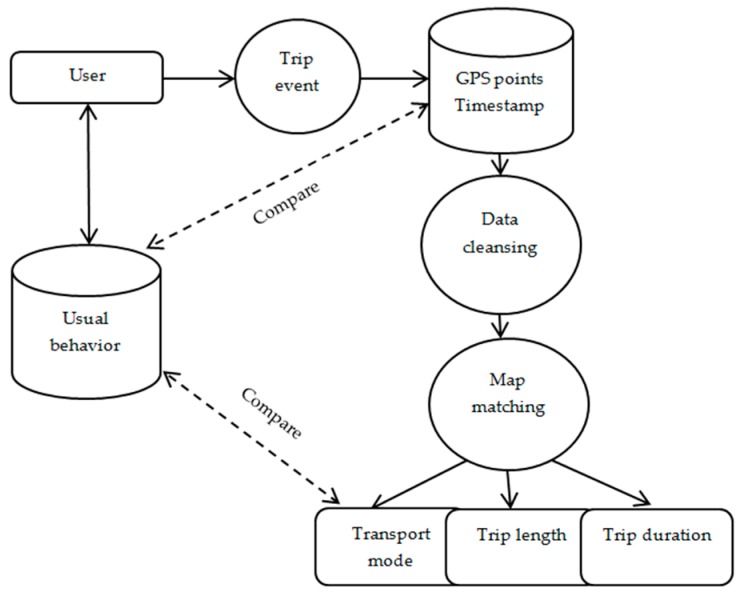
Data processing chain.

**Figure 8:**
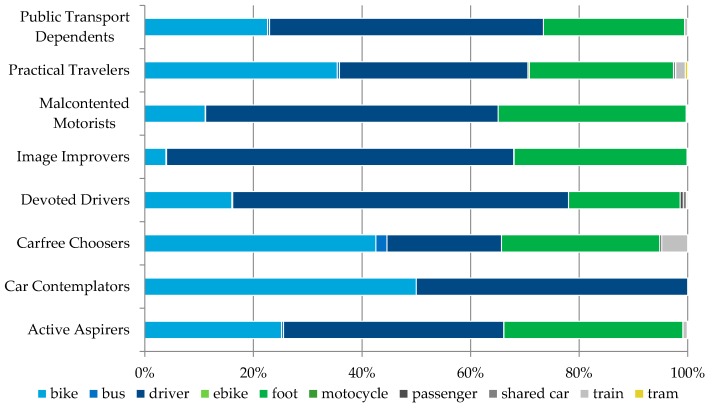
Modal split per attitudinal profile.

**Figure 9:**
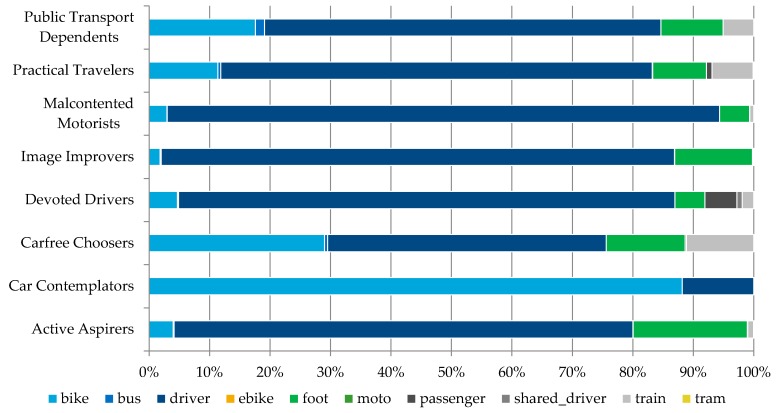
Modal split per kilometers travelled per attitudinal profile.

Every time a user would undertake a new trip the trip data were stored in the database and compared with the users’ usual behavior and mode choice (as defined through the web portal). To ensure reliability of the reported data, for every reported trip a Global Positioning System (GPS) location and a timestamp were collected with a frequency of 1 Hz. These data were used to confirm that the reported route and the transportation modes were equal to the observed ones (Figure 7). Based on the collected mobility behavior data we were able to deduct a detailed modal split for every attitudinal profile (Figure 8). By comparing the modal split with the modal split per travelled kilometer (Figure 9) one can see the tendency to use certain transportation modes for shorter or longer trips (e.g., Active Aspirers had tendency to make longer trips by foot than by bike).

## 4. Results

### 4.1. Suggestions Acceptance

**Figure 10:**
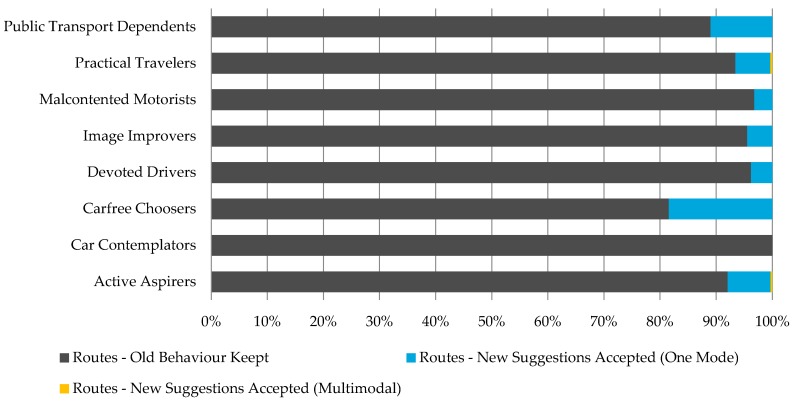
Acceptance of new route suggestions per attitudinal profile.

**Figure 11:**
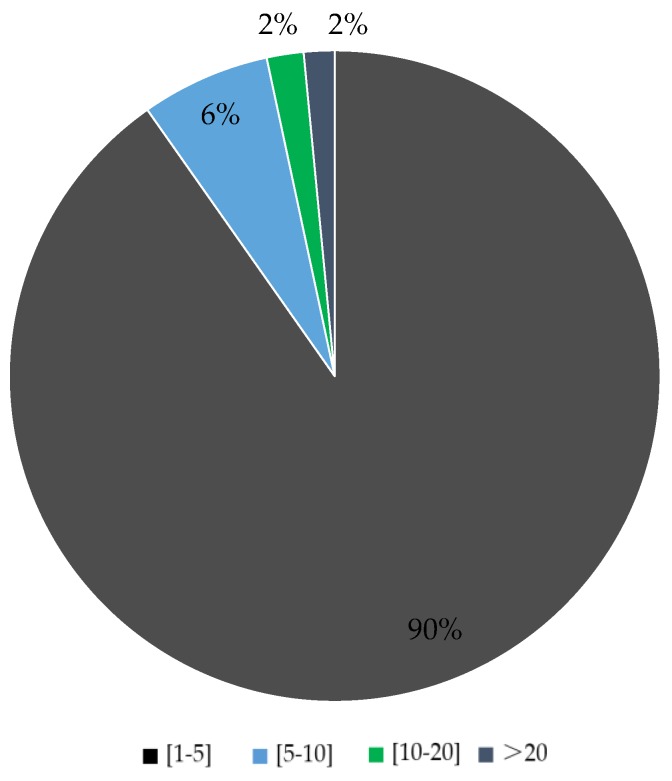
How many times the changed behavior was repeated.

By comparing the usual mobility behavior with the alternative route suggestions acceptance we were able to observe how keen members of each attitudinal profile are to accept alternative route incentives (Figure 10) and change their behavior. In addition, we observed that only Practical Travelers and Active Aspirers tried new multimodal routes while the rest have utilized single transportation mode options. Of all the observed behavioral changes (trips not made with usual mode choice) more than 40% were repeated multiple times, ranging up to repeating the alternative route suggestion 85 times (Figure 11).

**Figure 12:**
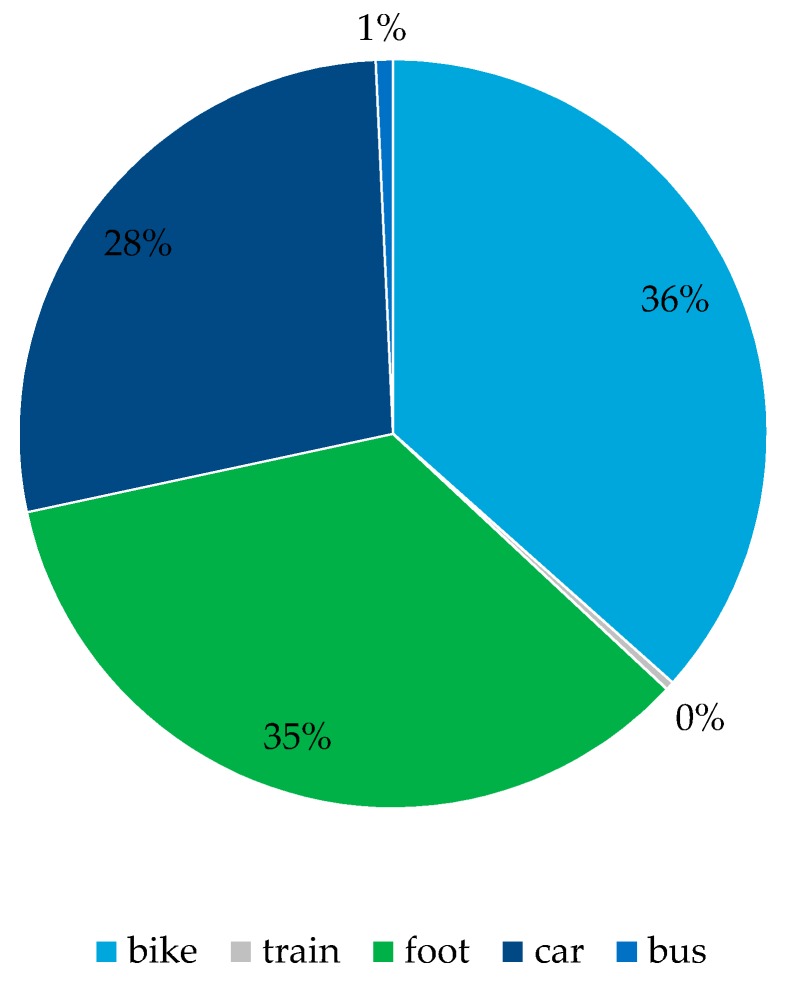
Overall—transportation modes for accepted suggestions.

**Figure 13:**
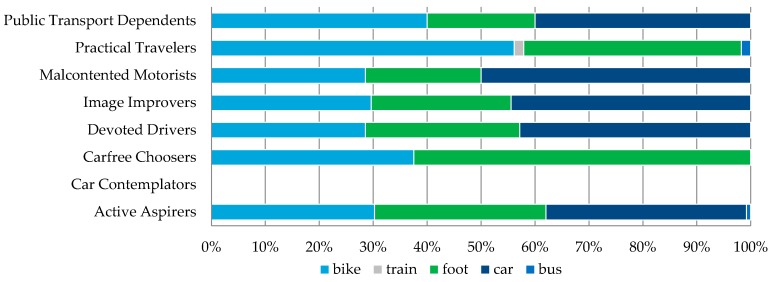
Modal split for taken suggestions per attitudinal segment.

Considering transportation modes, most often the alternative route suggestion involved biking or walking. In addition, faster routes by car or multimodal routes involving public transportation or train were also considered (Figure 12). The Practical Travelers had the most diverse modal split for considered alternative route suggestions (Figure 13).

### 4.2. Mode Choice Effects

**Figure 14:**
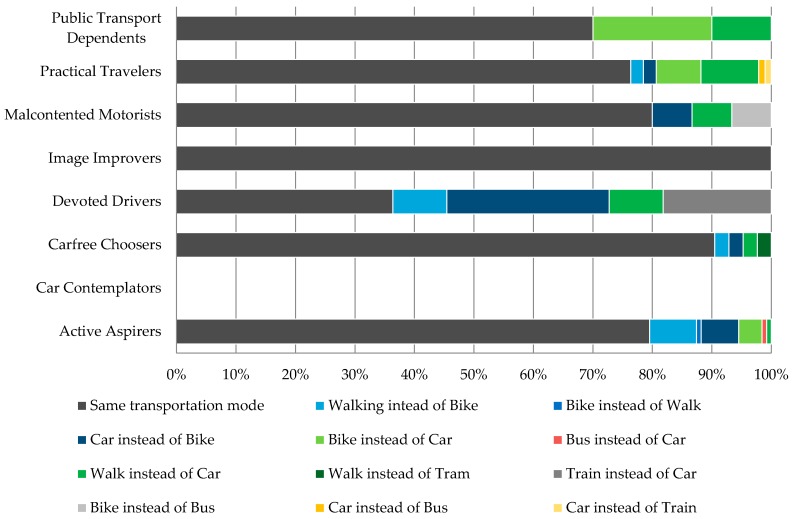
Observed modal shift.

When trying out alternative route suggestions, most often users utilized the faster route option with the same transportation mode as they would have usually (Figure 14). The Active Aspirers and the Practical Travelers were keener to try out alternative routes by transportation modes that they would not commonly use between defined origin and destination locations.

**Figure 15:**
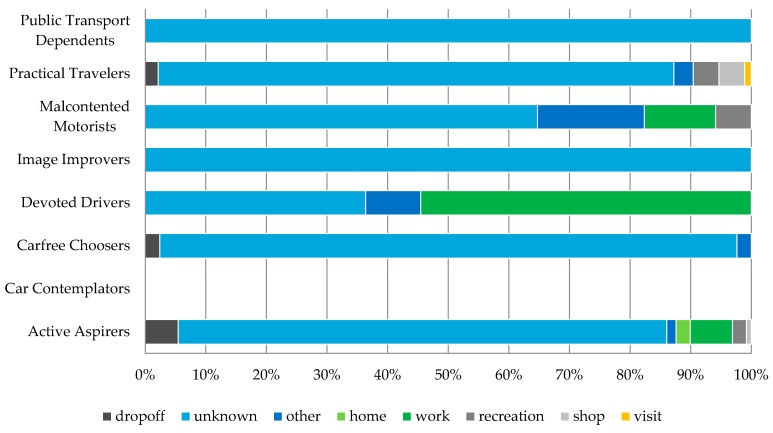
For what trip purposes new suggestions were accepted.

To analyze trip purposes for which users were most open to considering alternative route suggestions, we relied on the information provided by the users through the web portal. As provision of this information was not obligatory, most of the times it was left unfilled. In these cases the ‘unknown’ trip purpose (Figure 15) is reported. Nevertheless, it was informative to notice diversity between trip purposes for which each attitudinal profile reported that they tried out alternative routes suggestions (e.g., the Devoted Drivers were most likely to try out new commuting routes while the Practical Travelers were likely to try alternative route suggestions for everything but commuting).

## 5. Discussion

We have found that provision of alternative route suggestion incentives has a different impact on modal shift for different attitudinal profiles. In addition, the fact that users have repeated the changed mobility behavior multiple times shows the potential of mobile sensing-based mobility platforms to initiate behavioral changes and encourage more sustainable mobility behavior. This option is particularly interesting for smart city policy makers as a way to collect, but also deliver, personalized mobility incentives to users belonging to different attitudinal profiles and monitor the responsivity through observed behavioral changes.

Of all users Carfree Choosers were most likely to try out new route suggestions and did so for 18% of their usual origin destination pairs. Practical Travelers and Active Aspirers were the only ones to explore alternative multimodal routes. All attitudinal segments showed a tendency to keep utilizing the usual mode choice but were keen to try out new, faster, routes which also resulted in more sustainable overall behavior. This effect was particularly noticeable for car-oriented attitudinal profiles for whom even small changes in usual mobility behavior meant fewer kilometers travelled by car. This inevitably contributes to more sustainable mobility behavior at a community level as it results in less pollution. Ideally, the effect of behavioral change could be so significant that attitudinal profile would need to be redefined and number of users belonging to car-oriented profiles would be reduced, but this seems to be too ambitious at this early stage of smart city mobility management platform development. Although it is an intriguing and promising idea, multiple synchronized incentives (e.g., introduction of park & ride locations along routes frequently used by car oriented profiles or similar) would have greater potential to accomplish this. Nevertheless, the mobile sensed platform shows more potential then to be used just for delivery of route suggestion incentives as in such cases it would be able to provide insights on where those target locations are and to provide feedback on the effect of the introduced measures as changed behavior could be simultaneously detected. Regarding trip purposes, only Malcontented Motorists, Devoted Drivers and Active Aspirers reported trying alternative route suggestions for commuting while Active Aspirers were open to trying out new route suggestions for the most diverse range of trip purposes. These findings confirm expectations from previous studies that a provision of mobility-related information can initiate behavioral changes only if the communicated information appeals to the attitudes, values and motivators of the target group [30,48,49]. Furthermore, our findings confirm those of Nkurunziza et al. [12], who examined motivators and barriers for modal shift towards bike commuting, and found that addressing physical barriers alone is likely to have little impact and that perceived motivator variables are strongly associated with bicycle commuting. In addition, when compared with results from literature [17] where traditional communication channels were used to deliver targeted messages, and in average for 4.5% of trips users have tried out alternative options, by using smartphones to deliver personalized message almost twice more behavioral changes were observed. Although, penetration rates for smartphones are growing, it is evident that their distribution is not uniform across the globe and among different socio-economic groups [50], however such results show great potential in the way innovative technologies can be used for smart city mobility management options. The literature suggests that policies which aim to influence car usage should be targeted at the market segments that are most motivated to change and willing to reduce frequency of car use [10]. Therefore a smart city policy platform, that implements mobile sensed data and attitude-based segmentation, seems particularly useful in this sense as it confronts policy makers with observed behavior so that effect of such policies can be immediately evaluated. The impact of innovative sensing options, and fast feedback they provide on policy making, especially in the mobility domain where planning time horizons are approximately 15–20 years long [51], needs to be further investigated.

Overall, although our sample included more than 3400 users belonging to the different attitudinal segments the same level of conclusions cannot be drawn for each of them. The Car Contemplators participated in the sample in a very low number (only four persons) and accepted none of the suggestions for alternative routes. As this subsample is very small we cannot conclude that Car Contemplators are insensitive to the provided incentive but rather to suggest investigating this topic in more details in future research on a wider sample. In addition, observed transportation mode shifts were not always the most desired ones and some users have replaced active transportation modes with car. To get a broader picture on this matter, it stays to further investigate circumstances under which this occurred and potential relations to external elements such as bad weather conditions or various trip purposes (e.g., dropping-off multiple family members or weekly supply grocery shopping) for a given occasion. Furthermore, the Segment attitudinal segmentation approach has been widely tested across Europe, where our research is also situated, but future research on transferability of this approach in other cultures is desired.

Overall, this research: (i) explored the use of smartphones as smart city platform sensing devices and we found that smartphones can be effectively used for provision of alternative route suggestion incentives and for sensing resulting behavioral changes; (ii) we extend the current level of knowledge on transportation system users’ attitudinal profiles by providing detailed mobile sensed description of their observed mobility behavior. So far existing insights were exclusively based on paper surveys [17,18,19]. The literature confirms that paper travel surveys deviate systematically from actual travel behavior (e.g., users underreport short trips) [52,53,54,55]. In this context, mobile sensed insight seems particularly valuable as it provides high accuracy and high spatial and temporal resolution of mobility behavior; furthermore, while state-of-the-art literature reports [29,56] development of proof-of-concepts for smart city applications that incentivize and target shifts in mobility behavior (iii) we, to the best of our knowledge, have provided the first quantified insights into responsivity to incentives delivered through mobile phones. In addition, so far studies have been focused on one of attitudinal profiles or smaller portions of targeted population (e.g., new residents or students) [17,18,19] and have measured shift in their mobility behavior; In our study, (iv) the first empirical results on more sustainable mode choice tendencies for different attitudinal profiles, based on the use of smartphones for sensing of human behavior, over general population have been reported; In addition, (v) we illustrated the smart city platform potential for development of personalized mobility management campaigns and policies. To do so, we have examined the use of smartphones, on the one hand, as input sensing devices for mobility behavior and, on the other hand, as a means to deliver smart city platform suggestions. In our case, the smart city platform suggestions were alternative route suggestions, but the smart city platform potential should not be seen as limited only to provision of this information.

## 6. Conclusions

This paper presents the first insights into a Policy 2.0 platform intended to incentivize target shift in mobility behavior for different attitudinal profiles as defined under the European Segment project. We have found that the theory of market segmentation is applicable for sustainable mobility related social marketing and that provision of alternative route suggestion incentives has a different impact on modal shift for different attitudinal profiles. In addition, we have provided empirical results on the applicability of rational choice models in incentivizing behavioral change for highly habitual activity such as traveling. We have observed that Carfree Choosers were most likely to try out new route suggestions. Practical Travelers and Active Aspirers had the most diverse modal shifts and were open to trying out new route suggestions for the widest spectrum of trip purposes. In addition, we have identified future research needs in order to gain a deeper understanding of additional external elements’ impact on the behavioral change hoping to facilitate implementation of smart city mobility platforms in mobility management campaigns and policies development.

## Figures and Tables

**Table 1:** Statistics per segment.

Profile	Users	Trips	Trips Per User
Active Aspirers	194	9374	48.3
Car Contemplators	4	5	1.25
Carfree Choosers	10	2248	224.8
Devoted Drivers	49	1099	22.4
Image Improvers	100	4534	45.3
Malcontent Motorists	50	2078	41.6
Practical Travelers	196	8673	44.3
Public Transport Dependents	11	415	37.7

## Abbreviations

The following abbreviations are used in this manuscript:

CO_2_	Carbon dioxide
GPS	Global Positioning System
Hz	hertz
IoT	Internet of Things
PM	particulate matter
